# Targeting the HGF/c-MET pathway: stromal remodelling in pancreatic cancer

**DOI:** 10.18632/oncotarget.20822

**Published:** 2017-09-11

**Authors:** Srinivasa P. Pothula, Zhihong Xu, David Goldstein, Neil Merrett, Romano C. Pirola, Jeremy S. Wilson, Minoti V. Apte

**Affiliations:** ^1^ Pancreatic Research Group, South Western Sydney Clinical School, Faculty of Medicine, The University of New South Wales, Sydney, Australia; ^2^ Faculty of Medicine, The University of New South Wales, Sydney, Australia; ^3^ Ingham Institute for Applied Medical Research, Liverpool, NSW, Australia

**Keywords:** HGF/c-MET pathway, pancreatic cancer, stromal-tumor interactions, pancreatic stellate cells, orthotopic model

## Abstract

Stromal-tumor interactions in pancreatic cancer (PC) impact on treatment outcomes. Pancreatic stellate cells (PSCs) produce the collagenous stroma of PC and interact with cancer cells to facilitate disease progression. A candidate growth factor pathway that may mediate this interaction is the hepatocyte growth factor (HGF)/c-MET pathway. HGF is produced by PSCs and its receptor c-MET is expressed on pancreatic cancer cells. We studied the effects on PC progression of inhibiting the HGF/c-MET pathway in the presence and absence of a representative chemotherapeutic agent, gemcitabine. Using an orthotopic model of PC we have shown that “triple therapy” (inhibition of both HGF and c-MET combined with gemcitabine) resulted in the greatest reduction in tumor volume compared to each of the treatments alone or in dual combinations. Importantly, metastasis was virtually eliminated in mice receiving triple therapy. Our *in vivo* findings were supported by *in vitro* studies showing that the increase in cancer cell proliferation and migration in response to PSC secretions was significantly inhibited by the triple regimen. Our studies suggest that a combined approach, that targets tumor cells by chemotherapy while inhibiting specific pathways that mediate stromal-tumor interactions, may represent a novel therapeutic strategy to improve outcomes in PC.

## INTRODUCTION

Around 338000 new cases of pancreatic cancer (PC) are diagnosed worldwide each year and this devastating disease is set to become the second leading cause of cancer related deaths in the next decade [1]. Thus, there is an urgent need to develop new therapies to tackle this disease. PC is characterized by pronounced desmoplasia or dense collagenous stroma, which is now well acknowledged to have an intrinsic role in PC progression [2–4]. Our Group has demonstrated that this collagenous stroma is produced by activated pancreatic stellate cells (PSCs) [5]. Several preclinical studies have reported a close bidirectional interaction between pancreatic cancer cells and PSCs [6–10] which facilitates pancreatic cancer progression as evidenced by increased tumor growth as well as metastasis [7, 10–15].

Identifying the factors mediating the observed interaction between PSCs and PC cells may provide insights into novel therapeutic targets in PC [3, 15]. A candidate factor that has received some attention in recent years is the hepatocyte growth factor (HGF), which has been shown to have a role in the progression of PC [16, 17], particularly with respect to stromal-tumor interactions [14, 18]. We have demonstrated that HGF is secreted by cancer-associated human PSCs (hPSCs) [14, 19]. Binding of HGF to its transmembrane cell surface receptor c-MET, which is expressed on cancer cells activates several intracellular cell-signalling pathways that play a pivotal role in cancer cell proliferation and migration. We have also demonstrated that c-MET is present on the surface of endothelial cells, which potentiate PSC-endothelial cell interaction thus indicating a role in angiogenesis, and a consequent influence on metastatic spread [19]. HGF/c-MET binding has been reported to stimulate further production of HGF by mesenchymal cells, resulting in the formation of a feed-forward loop [20, 21]. Our recent work has shown that this pathway plays a critical role in cancer cell and endothelial cell functions [14, 19].

Using an orthotopic model of PC produced by injecting a mixture of cancer cells and cancer-associated human PSCs (hPSCs) into mouse pancreas [14], we recently published the effects of HGF inhibition (using a specific neutralizing antibody AMG102) and a representative chemotherapeutic agent, gemcitabine, as single agents and in combination, on PC progression. Effect of HGF inhibition was equivalent to gemcitabine in reduction of tumor volume but had a significantly greater effect on reducing metastasis. Indeed, gemcitabine alone failed to prevent metastasis, paralleling the clinical experience of patients receiving adjuvant gemcitabine developing metastases during treatment. We also observed that when gemcitabine was combined with HGF inhibition, the anti-metastatic effect of the latter was lost [14]. This may be a consequence of a gemcitabine-induced predisposition to increased stemness and aggressiveness of cancer cells, as has been shown by several other previous studies [14, 22–25]. However, another possible reason is that modulating only one arm (i.e. HGF) of the HGF/c-MET pathway, may not be sufficient to achieve an optimal anti-metastatic response.

In view of the above, it was of interest to determine the effects on PC of targeting both the ligand and its receptor (HGF and c-MET respectively), in the presence and absence of gemcitabine. The aim of the current studies was to determine the effects of *i*) HGF neutralizing antibody AMG102, *ii*) a small molecule inhibitor for c-MET (Compound-A) and *iii*) the chemotherapeutic agent gemcitabine, alone or in dual and triple combinations on PC progression, using both *in vivo* and *in vitro* approaches.

## RESULTS

### *In vivo* studies

### Effect of HGF/c-MET inhibition ± gemcitabine on tumor volume

**Figure 1:**
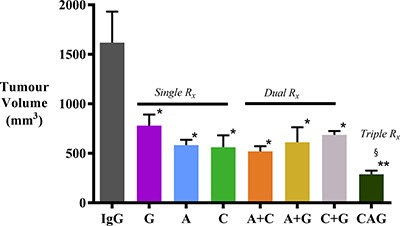
Effect of treatments on pancreatic tumor volumes Pancreatic tumor volumes were reduced to a similar extent by AMG102, compound-A and gemcitabine, whether administered as single agents or in dual combinations, compared to tumors in IgG treated (control) animals (*p < 0.005 vs IgG; *n* = 7/group). However, the greatest reduction of tumor volume was observed in mice treated with triple therapy (^§^*p* < 0.05 vs G; **p < 0.0001 vs IgG; *n* = 7/group). *Study Groups:* Control group (IgG/vehicle control). AMG102 - A, Compound-A (small molecule c-MET inhibitor)- C, Gemcitabine - G, AMG102 + Compound-A (A+C), AMG102 + gemcitabine (A+G), c-MET inhibitor + gemcitabine (C+G), AMG102 + c-MET inhibitor + gemcitabine (Triple therapy or CAG).

AMG102, compound-A, and gemcitabine as single agents significantly inhibited tumor growth when compared with IgG treated animals (Figure 1). Confirming our previously published results, HGF inhibition was as effective as gemcitabine in reducing tumor volumes in our model. Dual treatments did not exert any additive or synergistic effects on tumor volume reduction compared to single agents. However, the greatest reduction in tumor volume (to 17.9 ± 2.2 % of control mice receiving IgG treatment) was seen in the group of mice that received triple therapy (HGF inhibition + c-MET inhibition + gemcitabine).

### Effect of HGF/c-MET inhibition ± gemcitabine on tumor metastasis

**Figure 2:**
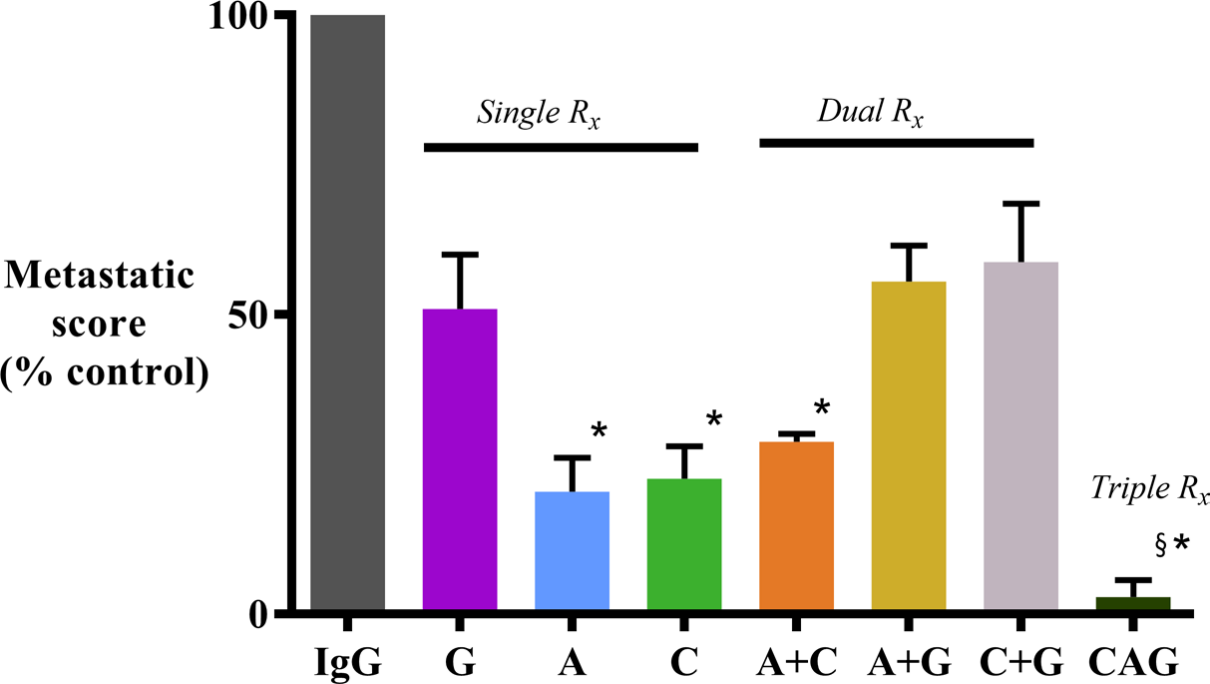
Effect of treatments on metastatic spread Mice treated with AMG102 and Compound-A (both as single agents and in combination) exhibited significantly reduced metastatic spread to the organs/regions depicted in the Table 1 when compared to IgG treated mice. In contrast, decrease in metastasis was not statistically significant in mice treated with gemcitabine as single agent or in dual combinations. Interestingly, mice treated with triple therapy exhibited virtual absence of metastasis. (*p < 0.05; ^§^*p* < 0.001 vs IgG, *n* = 7/group). *Study Groups:* Control group (IgG/vehicle control). AMG102 - A, Compound-A (small molecule c-MET inhibitor)- C, Gemcitabine - G, AMG102 + Compound-A (A+C), AMG102 + gemcitabine (A+G), c-MET inhibitor + gemcitabine (C+G), AMG102 + c-MET inhibitor + gemcitabine (Triple therapy or CAG)

Mice treated with AMG102 or Compound-A as single agents and in dual combinations exhibited significantly reduced metastasis when compared to mice receiving IgG (Figure 2 and Table 1). Mice treated with gemcitabine as a single agent or in combination with AMG102 or Compound-A displayed some reduction in metastasis but this change was not statistically significant when compared to IgG treated mice. In contrast, in mice receiving triple therapy, there was a *virtual elimination of metastasis* with only one mouse in the group displaying a solitary metastatic nodule in the liver (Figure 2 and Table 1). Metastatic score was calculated as number of visible nodules per site (liver kidney, retroperitoneum, bowel and diaphragm) x number of mice and expressed as % of control score in IgG treated mice.

**Table 1:** Metastatic nodules noted in number of mice treated in our orthotopic model in various sites (*n* = 7 mice/group)

	IgG	A	G	C	C+A	A+G	C+ G	C+A+G
**Liver**	7	2	6	3	2	5	6	1
**Kidney**	4	0	2	1	1	3	3	0
**Bowel**	7	2	3	1	2	4	3	0
**Retro-peritoneum**	7	2	3	1	2	2	4	0
**Diaphragm**	6	1	2	1	2	3	2	0
**TOTAL**	**31**	**7**	**16**	**7**	**9**	**17**	**18**	**1**

### Histological characterization of primary tumors

### Effect of treatments on cancer cell numbers

**Figure 3:**
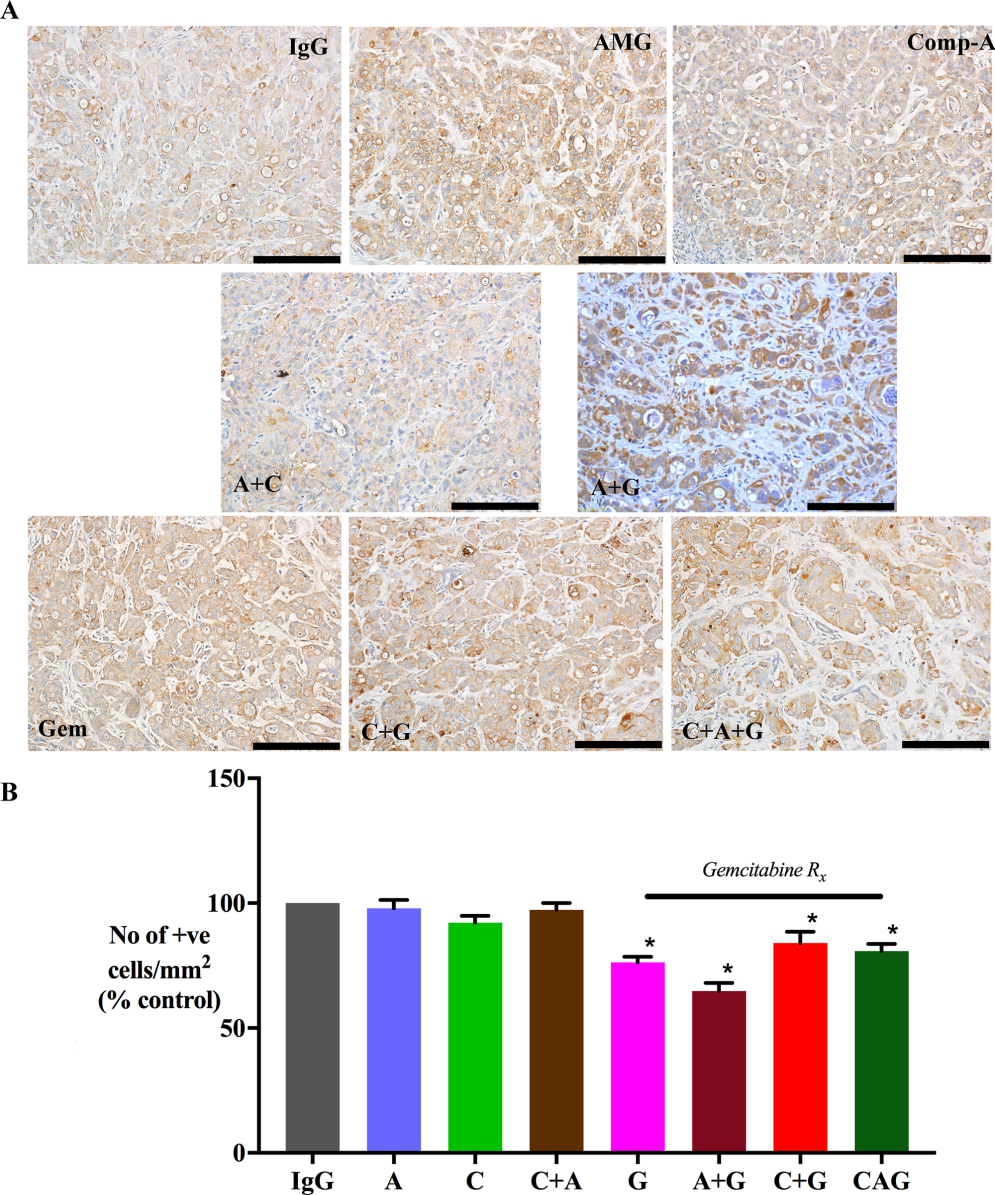
Effect of treatments on cancer cell density (**A**) Representative images of primary tumor sections immunostained for the cancer cell marker cytokeratin (scale bar: 50 μm). (**B**) A bar graph depicting morphometric analysis for cancer cell density [calculated as the number of brown stained cells per unit area of tumor, and expressed as % of control (IgG treated) tumors]. Compared to the IgG treated group, cancer cell density was significantly reduced in mice treated with gemcitabine singly and in dual or triple combinations (*p < 0.05 vs IgG, *n* = 7 mice/group), while AMG102 and compound-A as single and dual combinations failed to decrease cancer cell numbers compared to IgG controls. *Study Groups:* Control group (IgG/vehicle control). AMG102 - A, Compound-A (small molecule c-MET inhibitor)- C, Gemcitabine - G, AMG102 + Compound-A (A+C), AMG102 + gemcitabine (A+G), c-MET inhibitor + gemcitabine (C+G), AMG102 + c-MET inhibitor + gemcitabine (Triple therapy or CAG)

Morphometric analysis of tumor sections immunostained for cytokeratin allowed calculation of cancer cell density in tumors from each treatment group (expressed as number of cytokeratin positive cells/mm^2^). AMG102 and Compound-A treatments both as single agents as well as in dual combinations did not result in reduction of cytokeratin expression compared to IgG treated tumors (Figure 3). However, Gemcitabine alone or in combination with AMG102 or compound-A significantly reduced cytokeratin positive cells compared to IgG treated tumors (Figure 3). Mice treated with triple therapy exhibited a reduction in cancer cell numbers to a similar extent as gemcitabine treated groups and to a significantly less extent than in control mice (IgG treated mice). This effect is not unexpected given the cytotoxic effect of gemcitabine on cancer cells in these tumors.

### Effect of treatments on collagen deposition

**Figure 4:**
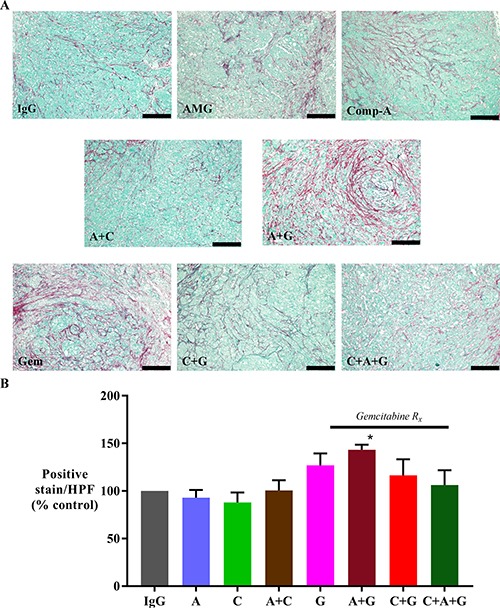
Effect of treatments on collagen deposition (**A**) Representative images of tumor sections stained for Sirius red (marker for collagen deposition) (scale bar: 200 μm). (**B**) A bar graph depicting the morphometric analysis for collagen deposition in our orthotopic model. Fibrillar collagen deposition was increased in animals treated with gemcitabine + AMG102 when compared to any other treatment (*p < 0.05 vs IgG, AMG102 and compound-A). *Study Groups:* Control group (IgG/vehicle control). AMG102 - A, Compound-A (small molecule c-MET inhibitor)- C, Gemcitabine - G, AMG102 + Compound-A (A+C), AMG102 + gemcitabine (A+G), c-MET inhibitor + gemcitabine (C+G), AMG102 + c-MET inhibitor + gemcitabine (Triple therapy or CAG)

The effect of treatments on fibrosis was assessed by software-assisted morphometric analysis on sections stained for Sirius red (which stains fibrillar collagen). AMG102 treatment alone did not change collagen deposition. Tumors from mice, which received AMG102+gemcitabine exhibited significantly higher Sirius Red expression than IgG treated mice (Figure 4). Tumors from mice treated with gemcitabine alone, and in combination with compound-A and with triple therapy also showed an increasing trend to higher Sirius red expression than IgG control mice but these increases did not reach statistical significance.

### Effect of treatments on cancer stem cell and EMT markers *in vivo*

The paradoxical lack of an anti-metastatic effect with gemcitabine treatment both as a single agent and in dual combination with HGF or c-MET inhibitor (Figure 2) suggested that gemcitabine treatment may be selecting out a sub-population of cancer cells, possible stem-like cells with an aggressive phenotype and increased migratory potential due to an increase in epithelial-mesenchymal transition (EMT) as occurred with our previous studies [14].

### i) Stem cell marker

**Figure 5:**
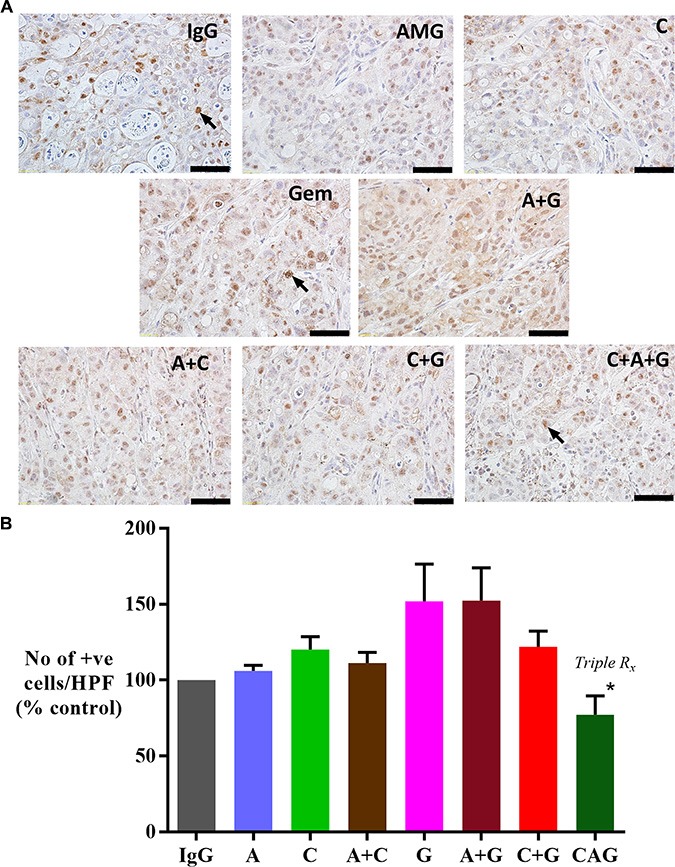
Effect of treatments on DCLK1 (stem cell marker) expression (**A**) Representative images of tumor sections immunostained for the stem cell marker DCLK1. Arrows indicate representative positive cells. (scale bar: 100 μm). (**B**) Graph depicting morphometric analysis for DCLK1 positive cells (number of brown positive cells per HPF, and expressed as % of control, IgG treated tumors). Compared to IgG treated tumors, there is a trend towards an increased number of cells expressing DCLK1 in gemcitabine treated mice when used as a single agent (G) or in combination with AMG102 (A+G). However this increase did not reach statistical significance. Notably, there is a significant reduction in expression of DCLK1 in tumors of mice treated with triple therapy when compared with other treatment involving gemcitabine as well as compound-A as single agent (*p < 0.02 vs G, A+G, C+G, C; *n* = 7 mice/group). *Study Groups:* Control group (IgG/vehicle control). AMG102 - A, Compound-A (small molecule c-MET inhibitor)- C, Gemcitabine - G, AMG102 + Compound-A (A+C), AMG102 + gemcitabine (A+G), c-MET inhibitor + gemcitabine (C+G), AMG102 + c-MET inhibitor + gemcitabine (Triple therapy or CAG)

Immunohistochemistry of primary tumor sections for the stem cell marker (DCLK1) demonstrated that tumors from mice treated with gemcitabine alone, or in dual combinations with either AMG102 or compound-A exhibited higher expression of DCLK1 (Figure 5). However, gemcitabine in combination with HGF+c-MET inhibition (triple therapy) resulted in a significant reduction in DCLK1 expression back to control levels.

### ii) EMT marker

**Figure 6:**
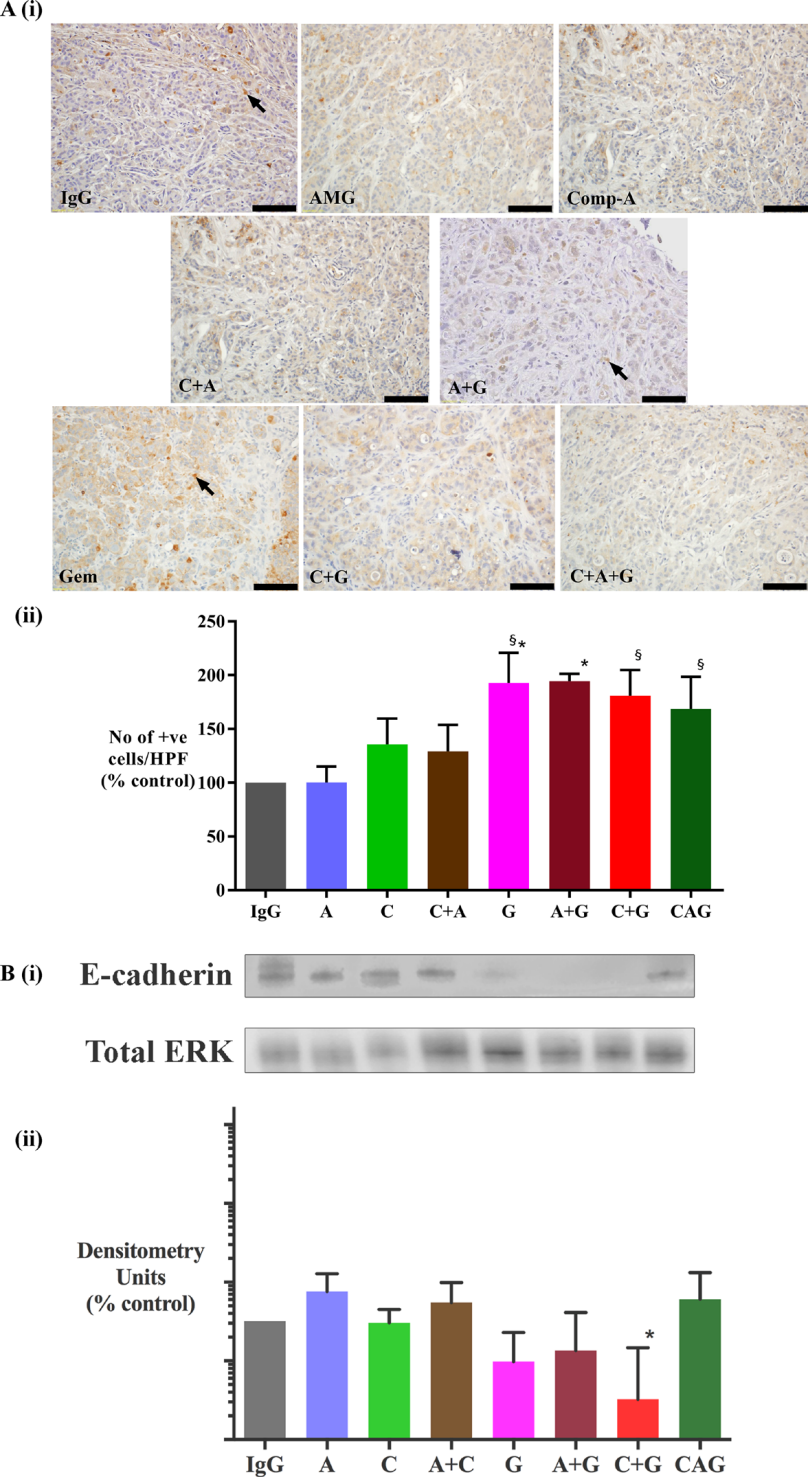
Effect of treatments on epithelial-mesenchymal transition (EMT) (**A**) Effect of treatments on TWIST (EMT marker) expression (i) Representative images of tumor sections immunostained for the EMT marker TWIST. Arrows indicate representative positive cells. (scale bar: 100 μm). (ii) Graph depicting morphometric analysis for TWIST positive cells (EMT marker) [number of brown positive cells per unit area of tumor, and expressed as % of control (IgG treated tumors)]. Compared to IgG treated tumors, the number of cells undergoing EMT was significantly increased by gemcitabine treatment both as a single agent (G) and in combination with AMG102 (A+G). Gemcitabine alone and in combination with compound-A as well as in triple therapy increased EMT when compared to AMG102 treated tumors (*p < 0.05 vs IgG, ^§^*p* < 0.05 vs AMG102, *n* = 7 mice/group). (**B**) Effect of treatments on E-cadherin (EMT marker) expression. (i) Representative immunoblots for e-cadherin (top panel) in tumor homogenates from the orthotopic model. The bottom panel shows immunoblots for total ERK, used as a loading control. (ii) Bar graphs showing densitometry results [corrected for respective loading controls and expressed as % of control in log_10_ scale (IgG treated tumors)]. Compared to control tumors, expression of e-cadherin is significantly reduced in mice treated with C+G. However this reduction is not seen in tumors from mice that received triple therapy (*^§^*p* < 0.05 vs A, A+C, CAG, *n* = 7 mice/ treatment group).*Study Groups:* Control group (IgG/vehicle control). AMG102 - A, Compound-A (small molecule c-MET inhibitor)- C, Gemcitabine - G, AMG102 + Compound-A (A+C), AMG102 + gemcitabine (A+G), c-MET inhibitor + gemcitabine (C+G), AMG102 + c-MET inhibitor + gemcitabine (Triple therapy or CAG).

Immunohistochemistry of primary tumor sections for the EMT marker (TWIST) demonstrated that tumors from mice treated with gemcitabine alone, or in combination with AMG102 exhibited significantly higher expression of TWIST compared to IgG treated control mice (Figure 6A). Although the mice receiving gemcitabine in combination with compound-A (C+G) as well as mice in the triple therapy groups demonstrated a trend to an increase in TWIST expression, this did not reach statistical significance. Immunoblotting tumor homogenates for e-cadherin showed a trend to a reduction in e-cadherin expression in tumors from mice treated with gemcitabine. This reduction was statistically significant in tumors from C+G group when compared with tumors from triple therapy group (Figure 6B).

### Effect of treatments on tumor angiogenesis *in vivo*

As HGF is known to have an angiogenic effect in several other cancers [26, 27], tumor sections were stained for the endothelial cell marker CD31, an indicator of neo-angiogenesis. Morphometric analysis of stained sections indicated a modest but statistically significant decrease in CD31 expression in tumors from mice treated with AMG102 compared to IgG treated tumors (82.63 ± 4.8% of IgG treated mice as control). These results are in keeping with our previous studies and concur with the proposed angiogenic role of HGF in cancers [14]. These data also support our previously published *in vitro* results, which demonstrated that HGF inhibition (using AMG102) directly reduced proliferation and migration of human microvascular endothelial cells, HMEC-1 [19]. However, c-MET inhibition alone or in combination with AMG102 did not affect CD31 expression (91 ± 6.2% and 87.6 ± 7% respectively). Gemcitabine treatment as a single agent or in dual or triple combination also did not result in any significant changes in CD31 expression (88.3 ± 7.4%, 95.5 ± 5%, 96 ± 6.4% and 89 ± 6% respectively).

### *In vitro* studies

### Role of HGF/c-MET in PSC-PC cell interactions *in vitro*

The role of the HGF/c-MET pathway in PSC-cancer cell interactions was determined by performing indirect co-culture experiments and assessing the effects of PSC secretions on cancer cell functions in the presence or absence of HGF/c-MET inhibitors and/or gemcitabine.

### i) Effect of HGF/c-MET inhibition ± gemcitabine on cancer cell proliferation *in vitro*

**Figure 7:**
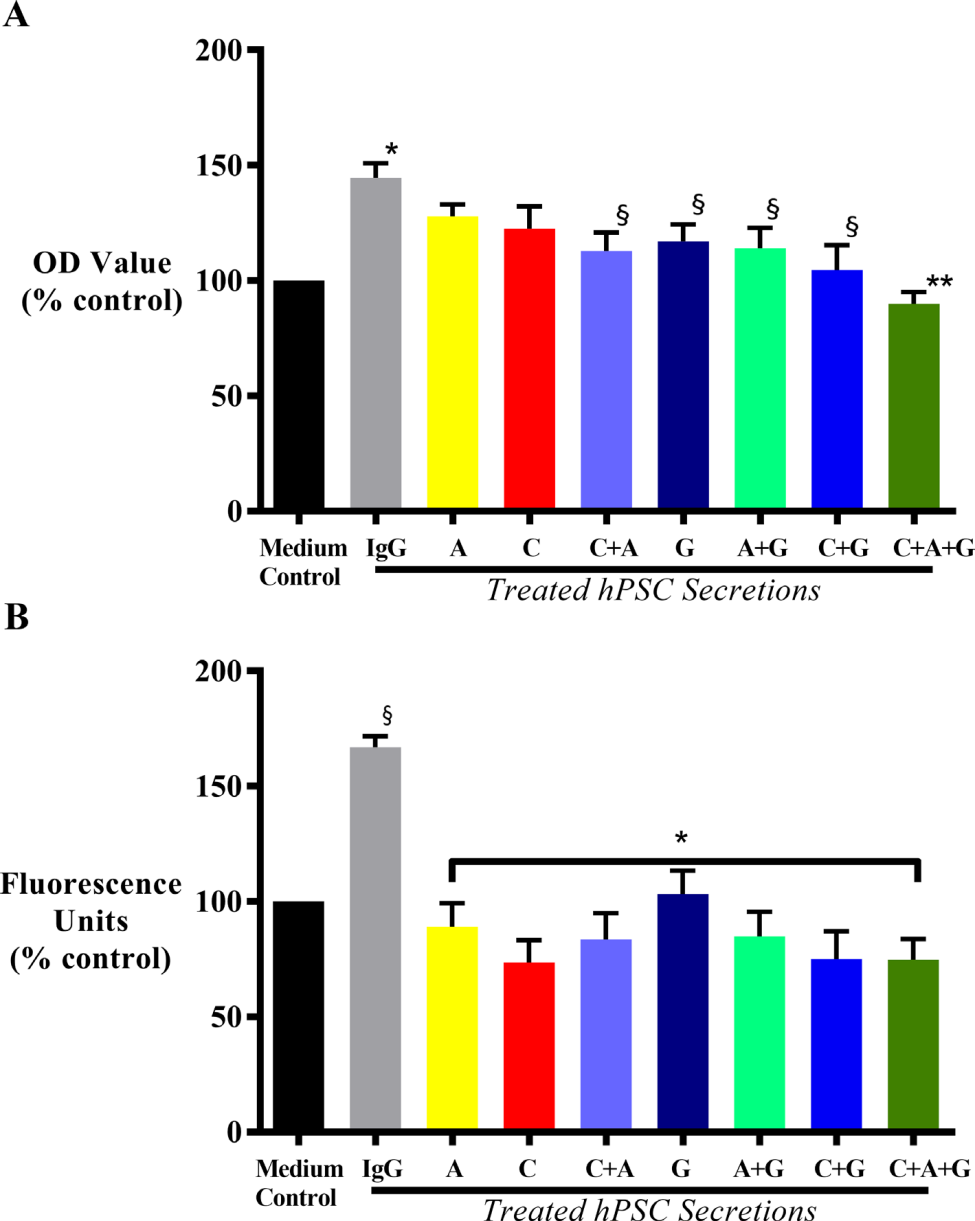
Effect of treatments on cancer cell proliferation and migration (**A**) Effect on cancer cell proliferation in response to treatment with HGF inhibitor AMG102 (A), c-MET inhibitor (C) or gemcitabine (G), alone or in dual or triple combination *in vitro.* AsPC-1 cells were incubated with medium control or IgG treated hPSC secretions, or hPSC secretions pre-treated either with AMG102, or c-MET inhibitor, or gemcitabine as single agents, or in dual or triple combinations. Compared to control, hPSC secretions (IgG group) significantly induced AsPC-1 proliferation (*p < 0.0001 vs medium control). This hPSC-induced AsPC-1 proliferation remained unchanged with either HGF (A) or c-MET inhibition (C) alone; but significantly inhibited with gemcitabine (G) as single agent and in any other combination (^§^*p* < 0.02 vs IgG). Similar reduction was also observed with HGF and c-MET inhibition as a dual combination (^§^*p* < 0.02 vs IgG). However, the greatest reduction was observed with triple therapy (C+A+G) in the treatment (**p < 0.0001 vs IgG; *n* = 5 separate hPSC preparations). (**B**) Effect on cancer cell migration in response to treatment with HGF inhibitor AMG102 (A), c-MET inhibitor (C) or gemcitabine (G), alone or in dual or triple combination *in vitro*. AsPC-1 cells were incubated with medium control or IgG treated hPSC secretions, or hPSC secretions pre-treated either with AMG102, or c-MET inhibitor, or gemcitabine as single agents, or in dual or triple combinations. Compared to medium control, hPSC secretions (IgG group) significantly induced AsPC-1 migration (^§^*p* < 0.0001 vs medium control). This hPSC-induced AsPC-1 migration was significantly inhibited with all the treatments (*p < 0.0001 vs IgG; *n* = 5 separate hPSC preparations).

hPSC secretions with known amounts of HGF (2000pg/mL) induced AsPC-1 proliferation compared to controls *i.e*. cancer cells incubated with co-culture medium alone, confirming our previously published studies [14, 28]. This hPSC-induced cancer cell proliferation was unchanged by exposure of cells to hPSC secretions pre-treated with AMG102 or when cancer cells were treated with c-MET inhibitor alone. However, hPSC-induced cancer cell proliferation was significantly inhibited by the combination of AMG102+c-MET inhibitor. Gemcitabine alone and in dual combinations also significantly reduced cancer cell proliferation. The greatest reduction in hPSC-induced AsPC-1 proliferation was observed with triple therapy (HGF inhibition + c-MET inhibition + gemcitabine) (Figure 7A).

### ii) Effect of HGF/c-MET inhibition ± gemcitabine on cancer migration *in vitro*

AsPC-1 cell migration was induced upon exposure to hPSC secretions, in agreement with our previously published reports [14, 28]. This induction of AsPC-1 migration was prevented by each of the treatments with the greatest reduction observed in AsPC1 cells exposed to the triple treatment (HGF inhibition + c-MET inhibition + gemcitabine) (Figure 7B).

### iii) Effect of HGF/c-MET inhibition ± gemcitabine on cancer cell apoptosis *in vitro*

For these studies, AsPC-1 cells were first subjected to serum starvation to induce apoptosis of the cells. In the presence of IgG treated hPSC secretions, AsPC-1 apoptosis induced by serum starvation was inhibited albeit to a modest level (94 ± 1% of medium control). However there was no effect on such apoptosis with HGF inhibition alone, gemcitabine alone or in their dual combination. Interestingly, AsPC1 apoptosis induced by serum starvation was modestly but significantly increased further in the presence of c-MET inhibition as a single agent or in dual combination with AMG102 or with gemcitabine (111 ± 2.5%, 108 ± 1.8%, 110 ± 3.5% of medium control respectively). Importantly, this pro-apoptotic effect persisted in cells treated with the triple combination (105.3 ± 2%).

### iv) Signalling pathways mediating the effects of HGF/c-MET inhibition on cancer cells

**Figure 8:**
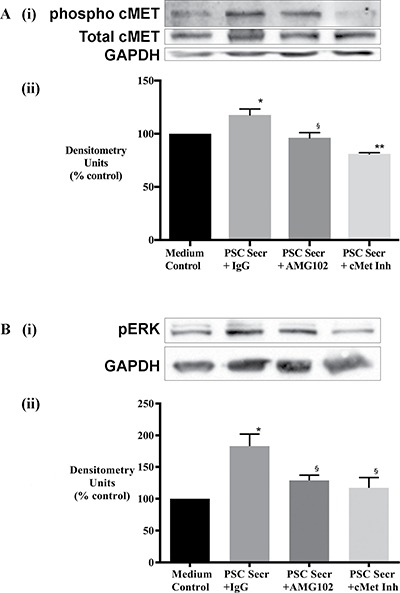
Effect of treatments on cancer cell signalling (**A**) Cancer cell response to HGF inhibition (AMG102) and c-MET inhibition (cMet Inh) was mediated by c-MET activation. (i) Representative immunoblots for phosphorylated (top panel) and total c-MET (middle panel) in AsPC-1 cell lysates collected after exposure of cells to hPSC secretions pretreated with either IgG or AMG102 or c-MET inhibitor (c-Met Inh) for 10 minutes. The bottom most panel shows GAPDH, used as a loading control. (ii) Bar graphs showing densitometry results [corrected for respective loading controls and expressed as % of control (co-culture medium)]. Compared to medium control, hPSC secretions significantly increased phospho-c-MET but not total c-MET (*p < 0.05, *n* = 3 hPSC preparations). This hPSC-induced phosphorylation of c-MET was inhibited with AMG102 (^§^p < 0.02 vs Secr+IgG, *n* = 3 hPSC preparations) as well as c-MET inhibition (**p < 0.01 vs Secr+IgG, *n* = 3 hPSC preparations). (**B**) Cancer cell response to HGF inhibition (AMG102) and c-MET inhibition (cMet Inh) was mediated by ERK1/2 activation. (i) Representative immunoblots for phosphorylated ERK1/2 and GAPDH in AsPC-1 cell lysates collected after exposure of cells to hPSC secretions pretreated with either IgG or AMG102 or c-MET inhibitor (c-Met Inh) for 15 minutes. The top panel shows phosphorylated ERK1/2 while the lower panel indicates GAPDH, used as a loading control. (ii) Graph showing densitometry results [calculated by correction for respective loading controls and expressed as % of control (co-culture medium)]. Compared to medium control, hPSC secretions significantly increased ERK1/2 phosphorylation (Secr+IgG; *p < 0.05 vs medium control). This hPSC-induced phosphorylation of ERK1/2 was inhibited with HGF inhibition and c-MET inhibition (^§^*p* < 0.01 vs Secr+IgG, *n* = 3 secretions from separate hPSC preparations).

To determine the effects of HGF/c-MET inhibition, c-MET phosphorylation and total c-MET were examined by immunoblotting. AsPC-1 cells were exposed to i) hPSC secretions pretreated with IgG (control) or with AMG102 (HGF antibody) and ii) hPSC secretions in the presence of PHA665752 (c-MET inhibitor). Immunoblotting showed increased phosphorylated c-MET as well as total c-MET in cancer cells exposed to hPSC secretions for 10 minutes. Phosphorylation of cMET was significantly reduced by c-MET inhibitor but not by HGF inhibition (Figure 8A), while expression of total c-MET did not show any significant reduction with either AMG102 or c-MET inhibitor.

Downstream ERK1/2 activation was also examined by immunoblotting. Increased phosphorylation of ERK was observed in cancer cells exposed to hPSC secretions for 15 minutes. This effect was reversed in the presence of the HGF antibody as well as the c-MET inhibitor (Figure 8B).

## DISCUSSION

Using both *in vivo* and *in vitro* approaches, this study has provided novel evidence to indicate that stromal reprogramming (by inhibiting the HGF/c-MET pathway) combined with chemotherapy significantly reduces pancreatic cancer progression. The most effective combination was ‘triple therapy’ comprising a standard chemotherapeutic agent (gemcitabine), a monoclonal antibody against human HGF (AMG102) and a c-MET inhibitor. This triple therapy induced significantly greater reduction in tumor volume compared to single and dual treatments and importantly, virtually eliminated metastasis in our orthotopic model of pancreatic cancer. Our *in vivo* findings with triple therapy were supported by the results of our *in vitro* experiments showing that triple combination inhibited both cancer cell proliferation and migration to a greater extent than other treatment combinations.

Both HGF inhibition and c-MET blockade (using the small molecule c-MET inhibitor Compound-A) alone resulted in tumor reduction to a similar extent as that observed with gemcitabine. Supporting these results, are our *in vitro* observations whereby HGF inhibition or c-MET blockade alone inhibited cancer cell proliferation. However, dual combinations i.e HGF inhibition + c-MET blockade, HGF inhibition + gemcitabine and c-MET blockade + gemcitabine did not exhibit any additive effect in terms of tumor reduction. Our findings with c-MET blockade + gemcitabine do not agree with studies that have reported a synergistic effect between inhibition of c-MET and gemcitabine [22, 29, 30]. Notably, none of these studies have considered the role of stromal cells in their preclinical cancer models. The most recent of these studies [29] has employed both syngeneic and xenogeneic orthotopic models to demonstrate the effects of c-MET inhibition in combination with gemcitabine: However the stromal cells employed for their *in vitro* studies were limited to endothelial cells and vascular smooth muscle cells, and lacked the predominant source of HGF i.e. PSCs. The discrepancy between the results of these studies and our data suggests that in the presence of a continuous source of HGF secretion (i.e. PSCs) as in our study, c-MET inhibition alone may not be sufficient to significantly reduce cancer progression.

The observed reduction in tumor volumes in treated mice could be a result of reduced cancer cell proliferation as well as increased apoptosis. In our *in vitro* experiments, serum starvation induced apoptosis in cancer cells; however in the presence of hPSC secretions this effect was inhibited and may explain the increased tumor volumes observed in IgG treated mice *in vivo*. However, gemcitabine and HGF inhibition alone or in dual combination did not influence cancer cell apoptosis; hence the reduced tumor volumes in these mice could be due to reduced proliferation rather than increased apoptosis of cancer cells. Interestingly, c-MET inhibition as a single agent and in all other combinations exerted a pro-apoptotic effect which is in keeping with similar published reports in other cancers [31–33]. This could be one of the mechanisms mediating the observed reduction in tumor volume observed with c-MET inhibition in our *in vivo* model.

With respect to fibrosis, tumors from mice that received gemcitabine treatment as a dual combination with HGF inhibition exhibited higher fibrosis compared to all other groups confirming our previous results [14]. Tumors from other mice that received gemcitabine also showed a trend towards increased (albeit not statistically significant) fibrosis. This could possibly be due to the increased activation state of PSCs in response to the factors released by gemcitabine affected cancer cells [14].

Several studies have linked activation of c-MET signalling to phosphorylation of intracellular signalling cascades such as PI3K/AKT, MAPK/ERK [34, 35] or FAK [36] in pancreatic cancer models, leading to tumor cell invasiveness, motility and resistance to gemcitabine therapy. In the current study, we observed that the effect of c-MET inhibition resulted in downregulation of ERK1/2 signalling, which concurs with our previous observation with HGF inhibition [14]. Effects of Akt signalling were not investigated as hPSC secretions did not induce phosphorylation of Akt as we previously reported [14]. Cancer cells exposed to hPSC secretions exhibited increased activation/phosphorylation of c-MET most likely in response to HGF in the secretions. As expected, this c-MET phosphorylation was prevented in the presence of the c-MET inhibitor PHA665752, as previously reported [32, 33, 37–39].

Regarding metastatic spread in our model, a significant reduction in metastasis was observed with c-MET inhibition as a single agent, as well as in dual combination with AMG102. However this anti-metastatic effect was lost when c-MET inhibition was combined with gemcitabine treatment. These observations are similar to our previous findings [14], where the anti-metastatic effect of AMG102 was lost when combined with gemcitabine. Taken together these observations strengthen our view [14], that gemcitabine selects out a population of cancer cells with enhanced survivability and migratory potential. We examined this concept by assessing orthotopic tumors for the expression of the stem cell marker double cortin like kinase-1(DCLK1), which is known to be elevated in PC and is associated with increased metastatic potential of the disease [43, 41] and the EMT marker TWIST.

While HGF/c-MET inhibition itself did not affect stemness or EMT, gemcitabine treatment alone as well as in combination with AMG102 or c-MET inhibitor induced both stemness as well as EMT as evidenced by increased expression of DCLK1 and TWIST, and reduced e-cadherin expression in the tumors. These observations support our previous findings [14] as well as others that indicate the formation of a chemo-resistant population of cancer cells following chemotherapy [23–25].

Important observations in this model were made in the mice that received all three agents (triple therapy). The greatest reduction of tumor volume was noted in these mice when compared to IgG treated mice. Notably, metastasis was virtually eliminated in these mice. Striking inhibition of metastasis despite the presence of gemcitabine was surprising since as noted above, gemcitabine alone or in dual combinations did not prevent metastasis and actually increased the stemness and the migratory potential of cancer cells. In contrast, when gemcitabine was part of the triple therapy regimen, stemness of cancer cells in the pancreatic tumors was seen to be significantly reduced.

With regard to EMT as assessed by e-cadherin expression, tumors of mice receiving gemcitabine as a single agent or in dual combination with HGF inhibition or c-MET inhibition exhibited reduction in e-cadherin expression, suggesting increased EMT. This concurs with the observed increase in metastasis in these groups. Interestingly pancreatic tumors of mice receiving triple therapy showed no such decrease in e-cadherin expression, suggesting that EMT was not increased in this group, thus supporting the observed absence of metastasis. However, this effect on EMT in the triple therapy group was not evident with another EMT marker, the transcription factor TWIST. The lack of an inhibitory effect on TWIST expression, despite the significant inhibition of metastasis, in these mice was unexpected. This observation raises the possibility that factors other than TWIST may regulate EMT in pancreatic cancer cells, warranting future work in this area.

**Figure 9:**
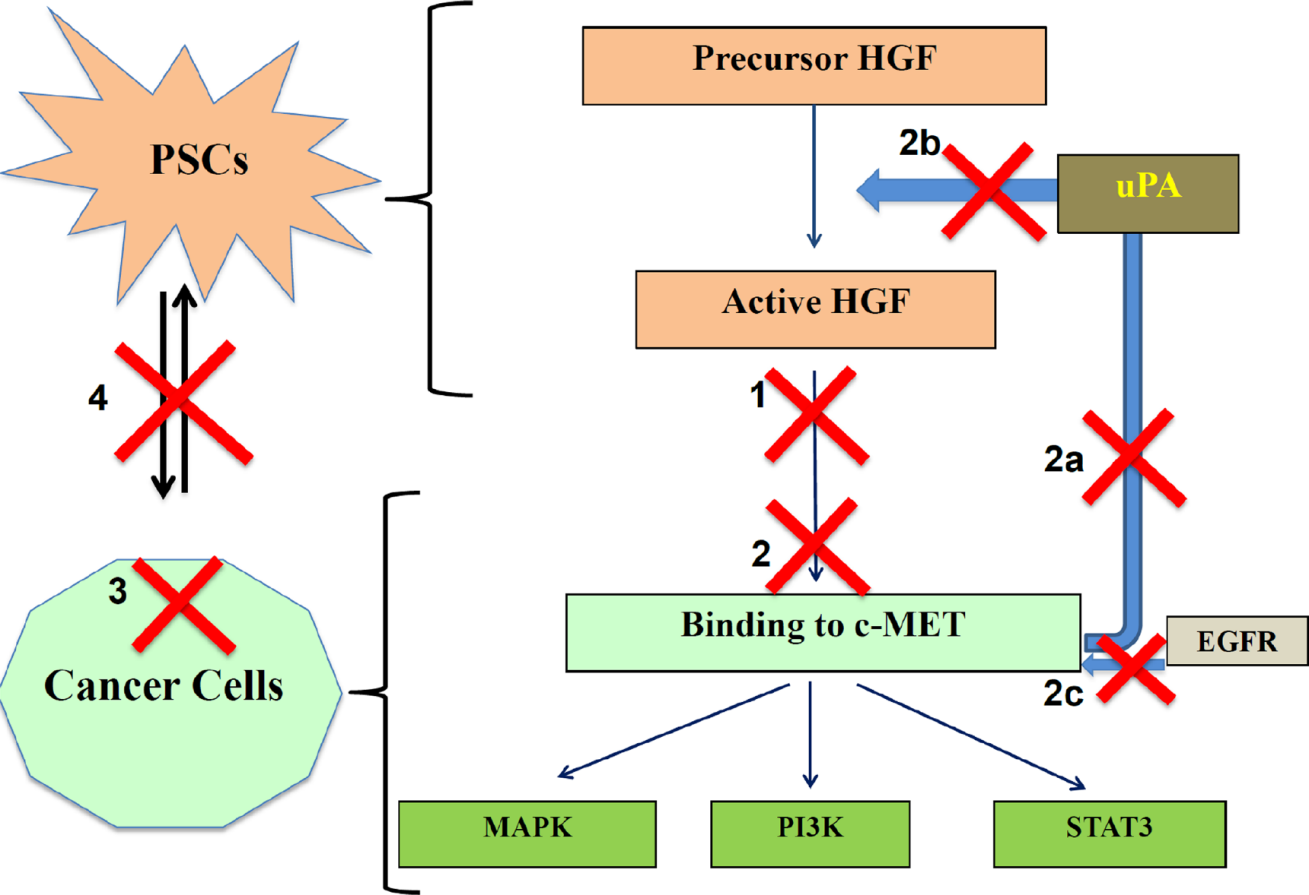
Schematic depicting the possible mechanisms involved in effects of triple therapy on PC progression Possible actions of these compounds are shown as numbered red crosses in the figure. 1. AMG102 neutralizes active HGF, reducing HGF/c-MET binding. 2. c-MET inhibitor works by preventing binding of ligand HGF to the receptor c-MET directly by inhibiting the pathway. Additionally, c-MET inhibitor acts by: 2a) Reducing uPA production by cancer cells. 2b) Inhibiting further HGF activation. 2c) Preventing transactivation of c-MET by other pathways such as EGFR. 3. Gemcitabine causes cancer cell death. 4. Overall the bidirectional interactions between PSCs and cancer cells are significantly reduced.

We postulate that the striking inhibitory effects of triple therapy on PC progression in our orthotopic model could be explained by a combination of effects that occur along the HGF/c-MET pathway. In untreated PC, stromal PSCs secrete HGF in its precursor form, which is activated by proteases such as urokinase plasminogen activator (uPA). Binding of HGF to its receptor c-MET on cancer cells stimulates several intracellular signalling cascades, which regulate cancer cell functions such as proliferation, migration and apoptosis. In addition, binding of HGF to c-MET increases production of uPA in cancer cells [42], which further activates precursor HGF to active HGF, thus forming a feed forward loop (Figure 9).

Three elements of triple therapy possibly inhibit several steps along the HGF/cMET pathway:

1) HGF inhibition neutralises PSC-derived active HGF in the ECM surrounding cancer cells, thus preventing HGF/c-MET binding. 2) c-MET inhibition blocks ligand-receptor binding leading to. a) inhibition of downstream intracellular signalling cascades. b) reduction of uPA production by cancer cells, thus preventing further HGF activation (thus inhibiting the positive feedback loop).c) blockade of stemness in cancer cells (given that c-MET is recognized to be a stem cell marker in pancreatic cancer [30, 43]). d) blockade of the transactivation of the c-MET receptor that is known to occur via other pathways such as the epidermal growth factor receptor (EGFR) [44].

3) Gemcitabine, a nucleoside analogue, causes cancer cell death [45].

The overall outcome of the above effects is to inhibit the well-established bidirectional interaction between PSCs and cancer cells in addition to killing cancer cells, thereby resulting in decreased tumor growth and metastasis.

In summary our study indicates that a novel and an effective therapeutic approach to pancreatic cancer may be achieved with a two-pronged approach that targets not only cancer cells but also stromal cells to interrupt stromal-tumor interactions that facilitate cancer progression. To date, a number of targeted therapies that showed significant promise in pre-clinical studies have failed to translate successfully to the clinical situation [46–49]. We believe this is partly due to the absence of a stromal component in these pre-clinical models. The use of a more biologically relevant model of pancreatic cancer involving both human pancreatic cancer cells and human pancreatic stellate cells increases the potential for our results to be translated to the clinical situation.

## MATERIALS AND METHODS

### Reagents

Reagents include Iscove's Modified Dulbecco's Medium, RePMI Medium 1640, fetal bovine serum, glutamine, and 4,6-diamidino-2-phenylindole (DAPI) from (Life Technologies Corporation, Tullamarine, VIC, Australia); Dulbecco's Modified Eagle's Medium and Xten Hybricell SFM Medium from Thermo Fisher Scientific (Waltham, MA, USA); anti–human alpha smooth muscle actin (αSMA) antibody, anti-human DCLK1 antibody, from Sigma-Aldrich (St. Louis, MO, USA); anti-human cytokeratin antibody, 3,3–diaminobenzidine (DAB) tetrahydrochloride substrate from DAKO (Campbellfield, VIC, Australia); anti-human CD31 antibody, anti-human TWIST antibody from Abcam (Melbourne, VIC, Australia) and anti- human glial fibrillary acidic protein (GFAP) antibody (Millipore, North Ryde, NSW, Australia); Safil 5/0 polyglycolic acid absorbable surgical suture from B. Braun (BellaVista, NSW, Australia); Cell culture inserts 8μM pores (Becton-Dickinson, Bedford, MA, USA); CytoSelect Tumor Transendothelial Migration Assay kit from Cell Biolabs, Inc. (San Diego, CA, USA); Cell Counting Kit-8 (Dojindo Technologies Pty Ltd, Parkville, VIC, Australia) multi-parameter apoptosis kit from Cayman chemicals, (Ann Arbor, MI, USA) Pierce Bicinchoninic Acid (BCA) protein assay kit (Thermo Fisher Scientific Pty Ltd, Scoresby, VIC, Australia). HGF neutralizing antibody, (AMG102/ Rilotumumab) and small molecule inhibitor for c-MET (Compound-A) from Amgen Inc. (Amgen Inc., Thousand Oaks, CA, USA), gemcitabine as gemcitabine hydrochloride from Hospira (Mulgrave, VIC, Australia), and c-MET inhibitor 100nM (PHA665752, Tocris Bioscience, Noble Park, VIC, Australia) were used. Antibodies for anti-e-cadherin (Abcam, Melbourne, VIC, Australia), anti-human phospho-p44/42 MAPK (ERK1/2) and total ERK1/2 (Cell Signaling Technology, Danvers, MA, USA) were used.

### Orthotopic model of pancreatic cancer (*in vivo* studies)

The orthotopic model used for these studies has been described in detail previously [7, 10, 14, 50]. Briefly, 6–8 week old female athymic nude mice (BALBc nu/nu) were anaesthetized and an incision made in the left flank followed by exteriorization of the spleen and tail of the pancreas. A mixture of human PC cells (AsPC-1) 1 × 10^6^ + human PSCs 1 × 10^6^ in 50 μL PBS was injected into the tail of pancreas. This ratio of PC cells to PSCs was chosen to replicate early cancer development and progression. Seven days after implantation of AsPC1+hPSCs, mice were randomized to receive treatment as detailed below for a further 6 weeks.

### Animal grouping (*n* = 7 mice/group)

Group 1 Control group (IgG/vehicle control). 200 μl PBS with isotype IgG twice weekly intraperitoneal (IP) injections and daily oral gavage of soybean oil as vehicle control for Compound-A (small molecule inhibitor for human c-MET) (Group referred to in figures as “IgG”) Group 2 AMG102 (monoclonal antibody against human HGF) dissolved in 200 μl PBS 300 μg IP twice weekly; dose based on our previously published studies [14, 18]. (Group referred to in figures as “A”) Group 3 Compound-A (small molecule c-MET inhibitor) 60 mg/kg BW dissolved in soybean oil administered as daily oral gavage; dose based on preliminary dose-response studies establishing the maximally effective and non-toxic dose. (Group referred to in figures as “C”) Group 4 Gemcitabine 75 mg/kg BW IP twice weekly (Group referred to in figures as “G”) Group 5 AMG102 + Compound-A (A+C) Group 6 AMG102 + gemcitabine (A+G) Group 7 c-MET inhibitor + gemcitabine (C+G) Group 8 AMG102 + c-MET inhibitor + gemcitabine (Triple therapy or CAG).

Pancreatic tumor growth was monitored by palpation. Mice were sacrificed after six weeks of treatment. Primary tumors were resected and their size measured using digital Vernier callipers by two separate observers. Tumor volume was calculated to two decimal points, according to an established formula [1/2 (length x breadth x width) [51]. Tumor tissue was then dissected out for further processing. The abdominal cavity, mesentery, spleen, liver and lungs were examined and scored according to the presence or absence of visible metastatic nodules. Metastatic nodules were collected, fixed in formalin and processed for H&E staining. Tumors were compared with respect to size and weight. Tumors were stained with H&E for morphology, while collagen deposition was assessed by Sirius red staining as described previously [10, 28]. Primary tumor sections were also immunostained for cytokeratin (cancer cell marker), DCLK1 (stem cell marker), TWIST (EMT marker), and CD31 (endothelial cell marker).

### Expression of αSMA, cytokeratin, CD31, DCLK1 & TWIST in primary tumors

Paraffin sections (4 μm thickness) of primary tumors were dewaxed and rehydrated. Following heat-mediated antigen retrieval, tumor sections were incubated overnight at 4°C with respective primary antibodies as described previously [14]. Immunostaining of tumor sections for αSMA (1:800), cytokeratin (1:75), CD31 (1:50), DCLK1 (1:500), and TWIST (1:500) were performed using anti-human primary antibodies in provided dilutions in blocking buffer. This was followed by incubation with respective HRP-labelled secondary antibodies. Subsequently, the sections were incubated with DAB substrate and the signal was visualized by chromogen.

For morphometric analyses, ten fields were selected randomly (by observers blinded to treatment groups) for each tissue section and positive stained (brown) cells were counted, as described previously [14, 28]. Endothelial cells in primary tumors were identified by immunostaining for the endothelial cell marker CD31. Cells expressing stem cells and EMT characteristics were assessed by immunostaining the tumor sections for stem cell marker DCLK1 and transcription factor TWIST respectively. Results were analysed as the total number of positive cells in all 10 selected fields and expressed as % of control (i.e. tumor sections from IgG treated mice).

### Immunoblotting on tumor homogenates

Tumor samples that were snap frozen were homogenized by mechanical disruption using a mortar and pestle in presence of liquid nitrogen in RIPA buffer with protease and phosphatase inhibitors. Proteins were quantified using bicinchoninic acid assay (BCA assay) according to Manufacturer's instructions. Homogenates were then subjected to immunoblotting as detailed below for assessment of e-cadherin expression.

Proteins were separated using 10% SDS polyacrylamide gels and transferred onto nitrocellulose membranes. Membranes were blocked for one hour in 5% skim milk in TRIS buffered saline with Tween-20 (TTBS), followed by overnight incubation at 4°C anti-rabbit e-cadherin 1:500. Following a wash protocol, membranes were incubated for one hour at room temperature with an HRP-conjugated goat anti-rabbit secondary antibody (DAKO Australia Pty Ltd, Kingsgrove, NSW, Australia), diluted 1:2000 in blocking buffer. Target proteins were detected using the Bio-Rad ECL kit (Bio-Rad, Philadelphia, PA, USA) as described previously [14]. Loading control total ERK1/2 was determined by incubating overnight at 4°C with anti-rabbit ERK1/2 antibody (Cell Signaling Technology, Danvers, MA, USA) diluted 1:1000 in TTBS. Densitometry readings for e-cadherin were corrected to their respective loading controls. Results are expressed as % of control (*i.e*. tumor homogenates from IgG treated tumors).

### *In vitro* studies: cell culture

AsPC1 cells were cultured and hPSCs were isolated and cultured as previously published by us [14].

### Indirect co-culture experiments

To study the role of the HGF/c-MET pathway in the interactions between PSCs and cancer cells *in vitro*, indirect co-cultures were set up which involved incubation of cancer cells with conditioned medium from PSCs.

### Collection of conditioned medium from PSCs and HGF measurement

As described previously, PSCs were passaged by trypsinization when 70–80% confluence was reached. Conditioned media (hPSC secretions) used for experiments were collected from hPSCs. Media (0.1% SFM4MAb for proliferation and migration assays or 0% IMDM for apoptosis assays) were collected, centrifuged at 1,000g for 10 min at 4°C, and the supernatant was concentrated with Centricon YM3 filters (Millipore). HGF in hPSC secretions was quantified using a human HGF Quantikine ELISA Kit (R&D Systems) according to the manufacturer's instructions as described previously [19]. The secretions were stored at –80°C until used for functional assays. For assays that required pretreated secretions, appropriate dilution of secretions was performed with co-culture medium so as to standardize HGF to a concentration of 2000pg HGF/ml. This concentration was used since it reflects the actual amount produced by hPSCs over 24 hours [14, 19].

### Pretreatmeant of hPSC secretions and cancer cells

Secretions from hPSCs (*n* = 5) that had been standardized to 2000 pg HGF/mL were pretreated for one hour at 37°C with either 60 μg/mL AMG102 (HGF neutralizing antibody) or 60 μg/mL IgG (isotype control for AMG102). Similarly cancer cells were pre-exposed to the c-MET inhibitor 100 nM (PHA665752) for one hour at 37°C. Following this pretreatment of cancer cells, hPSC secretions ± gemcitabine (300 μg/mL) were added to the cells. Therefore the experimental set up included cancer cells treated with PSC secretions in presence and absence of either AMG102 or c-MET inhibitor or gemcitabine.

The concentrations of HGF antibody, c-MET inhibitor and gemcitabine were calculated to correspond to the *in vivo* doses used in these studies, assuming the total circulating volume of a 20 gm mouse to be 5mL as reported in our previous publication [14].

*Note: For our in vitro studies a commercially available c-MET inhibitor (PHA665752) that has been widely used in published literature was employed* [37, 38] *since it is water soluble. Compound-A used in our in vivo model, is soluble only in soybean oil and therefore could not be used in culture medium.*

### Proliferation of AsPC-1 cells in response to indirect co-culture

The effects of HGF inhibition on AsPC-1 proliferation were measured using the Cell Counting Kit-8. AsPC-1 cells were seeded at a density of 5000 cells/well in a 96 well plate. The following day, culture medium was removed and wells were rinsed twice with warm PBS. 200 μL of pretreated hPSC secretions (see above) were applied to each well. After incubation for 24 hours at 37°C the assay was performed according to Manufacturer's instructions. This assay uses a tetrazolium salt, which produces a water-soluble formazan when reduced. The amount of formazan produced is directly proportional to the number of living cells. The optical density of the wells was determined at 450 nm.

### Migration of AsPC-1 cells in response to indirect co-culture

AsPC-1 cell migration was assessed using a modified Boyden chamber method as published by our Group previously [10, 52] according to Manufacturer's instructions. Number of migrated cancer cells was assessed by recording fluorescence (480 nm/520 nm) on the plate using a SpectraMax M2e micro plate reader (Molecular Devices, Sunnyvale, CA, USA).

### Apoptosis of AsPC-1 cells in response to indirect co-culture

AsPC-1 cells were seeded at a density of 15,000 cells/well in a 96-well opaque plate and cultured in serum-free IMDM overnight to induce apoptosis. Cells were then incubated with hPSC secretions (pre-treated as described above) for 24 hours and apoptosis was assessed by staining for cell based Annexin–V tagged with FITC as per the Multi-parameter Apoptosis Kit instructions (Cayman Chemicals, Ann Arbor, MI, USA). Apoptosis was assessed by recording fluorescence (485 nm/535 nm) on the plate using a SpectraMax M2e micro plate reader (Molecular Devices, Sunnyvale, CA, USA).

### Immunoblotting for signalling pathways (c-MET phosphorylation and MAPK activation)

AsPC-1 cells were exposed to one of the following treatments for 15 minutes: i) co-culture medium; ii) hPSC secretions + IgG; iii) hPSC secretions + AMG102, iv) hPSC secretions + c-MET inhibitor (as described above, using secretions from *n* = 3 different hPSCs preparations). After 15 minutes exposure, secretions were removed, cells were lysed and protein quantified using bicinchoninic acid assay (BCA assay) according to Manufacturer's instructions. Lysates were then subjected to immunoblotting as detailed below for assessment of MAPK activation. Similarly lysates from AsPC-1 cells were collected for assessment of c-MET activation after treatment for 10 minutes.

Lysed proteins were separated using 10% SDS polyacrylamide gels and transferred onto nitrocellulose membranes. Membranes were blocked for one hour in 5% skim milk in TRIS buffered saline with Tween-20 (TTBS), followed by overnight incubation at 4°C anti-rabbit phospho-MET (Tyr1234/1235) (D26, Cell Signaling Technology, Danvers, MA, USA) 1:1000, anti-rabbit total MET (D1C2, Cell Signaling Technology, Danvers, MA, USA) 1:1000 and anti-mouse GAPDH (Abcam, Melbourne, VIC, Australia) 1:50000. Overnight incubation with phospho-p44/42 MAPK (ERK1/2) (Cell Signaling Technology, Danvers, MA, USA) rabbit mAb diluted 1:1000 in TTBS with 5% BSA (bovine serum albumin) was performed to determine MAPK activation. Following a wash, membranes were incubated for one hour at room temperature with an HRP-conjugated goat anti-rabbit secondary antibody (DAKO Australia Pty Ltd, Kingsgrove, NSW, Australia), diluted 1:2000 in blocking buffer. Target proteins were detected using the Bio-Rad ECL kit (Bio-Rad, Philadelphia, PA, USA) as described previously [14]. Loading control GAPDH was determined by incubating overnight at 4°C with anti-mouse GAPDH antibody (Abcam, Melbourne, VIC, Australia) diluted 1:50000 in TTBS. Densitometry readings for phosphorylated ERK1/2 and c-MET were corrected for their loading controls. Results are expressed as % of control (*i.e*. expression in cells treated with coculture medium as control).

### Statistical analysis

Data are expressed as mean ± SEM. Student's *t*-test, one-way analysis of variance with Tukey's post hoc test, or Fisher's exact tests were applied as appropriate. Analyses were performed using GraphPad Prism 6.00 for Mac OS X (GraphPad Software, San Diego, CA, USA).
